# Genetic Engineered Ultrasound-Triggered Injectable Hydrogels for Promoting Bone Reconstruction

**DOI:** 10.34133/research.0221

**Published:** 2023-09-01

**Authors:** Zhenyu Zhao, Huitong Ruan, Aopan Chen, Wei Xiong, Mingzhu Zhang, Ming Cai, Wenguo Cui

**Affiliations:** ^1^ Department of Orthopaedics, Shanghai Tenth People's Hospital, School of Medicine, Tongji University, No.301 Middle Yanchang Road, Shanghai 200072, China.; ^2^ Department of Orthopaedics, Shanghai Key Laboratory for Prevention and Treatment of Bone and Joint Diseases, Shanghai Institute of Traumatology and Orthopaedics, Ruijin Hospital, Shanghai Jiao Tong University School of Medicine, 197 Ruijin 2nd Road, Shanghai 200025, China.; ^3^ Department of Foot and Ankle Surgery, Beijing Tongren Hospital, Capital Medical University, 1 Dongjiao Minxiang, Beijing 100730, China.

## Abstract

Genetic engineering technology can achieve specific gene therapy for a variety of diseases, but the current strategy still has some flaws, such as a complex system, single treatment, and large implantation trauma. Herein, the genetic engineering injectable hydrogels were constructed by ultrasonic technology for the first time to realize in vivo ultrasound-triggered in situ cross-linking and cell gene transfection, and finally complete in situ gene therapy to promote bone reconstruction. First, ultrasound-triggered calcium release was used to activate transglutaminase and catalyze the transamidation between fibrinogen. Simultaneously, liposome loaded with Zinc-finger E-box-binding homeobox 1 (ZEB1) gene plasmid (Lip-ZEB1) was combined to construct an ultrasound-triggered in situ cross-linked hydrogels that can deliver Lip-ZEB1. Second, ultrasound-triggered injectable hydrogel introduced ZEB1 gene plasmid into endothelial cell genome through Lip-ZEB1 sustained release, and then acted on the ZEB1/Notch signal pathway of cells, promoting angiogenesis and local bone reconstruction of osteoporosis through genetic engineering. Overall, this strategy provides an advanced gene delivery system through genetic engineered ultrasound-triggered injectable hydrogels.

## Introduction

Genetic engineering technology provides powerful means for the research and application of gene structure and function, realizing efficient coordination of tissue regeneration and control of cell behavior [1–3]. These gene therapy strategies play a role in gene therapy of various diseases by accurately regulating various target genes formed in tissues [4,5]. Currently, a variety of gene delivery vectors such as nanoparticles, 3-dimensional (3D) printing scaffolds, and hydrogels can be combined with genetic engineering strategies to achieve in situ gene therapy for a variety of diseases [6–10]. For example, nanoparticles were used to load 2 growth factor plasmids and bind to sutures to integrate the 2 growth factor genes into injured tendon tissues to promote healing [11]. However, in this study, nanoparticles promoted tendon reconstruction by introducing 2 segments of target genes respectively, which made the construction of a genetic engineered plasmid and system more complicated and the expression stability was low. In addition, the combination of hydrogels and plasmids carrying vascular endothelial growth factor genes has been shown to accelerate burn wound healing and promote microangiogenesis [12]. However, the effect of single growth factor gene plasmid on vascular regeneration was low when introduced into endothelial cells. Recently, Moncal et al. [13] demonstrated the osteogenic bio-ink of BMP2 plasmid DNA, which promoted bone repair through 3D-printed scaffolds. However, the gene therapy of this scheme has a large wound when the material is implanted, which is highly likely to cause secondary damage, and it is difficult to achieve minimally invasive treatment. Therefore, there are still many defects in the existing strategies of nanoparticle, 3D printing scaffold, hydrogel, and other vectors combined with gene therapy to promote tissue regeneration, such as a complex plasmid system, single gene therapy approach, and the inability to conduct in situ minimally invasive treatment. Therefore, in the field of bone reconstruction, it is difficult to achieve advanced genetic engineering to promote bone regeneration.

At present, in order to effectively break through the bottleneck of gene therapy to promote tissue reconstruction, a variety of trigger technologies, such as light, magnetic, electrical, acoustic, etc., have been extensively combined with genetic engineering [14–19]. However, light penetration to deeper tissues is weak and its intensity is limited. Magnetic field trigger intensity is difficult to accurately control. Electric field and electrotherapy have low biological safety and high side effects [20,21]. Therefore, at present, the combination of magnetic, electric, light, and other trigger technologies and genetic engineering still cannot meet the needs of gene therapy to promote tissue regeneration. In recent years, ultrasonic triggering technology has emerged as an exciting and unique therapeutic approach that can track drug delivery; control treatment in dose, space, and/or time; and improve drug delivery efficiency with high spatial precision [22–24]. Ultrasonic triggering technology combined with biological materials, including hydrogels, microvesicles, nanovesicles, liposomes, micelles, etc., can meet the gene therapy needs of different tissues, organs, and diseases from different angles [25–27]. For example, ultrasound-triggered microvesicles enhance the efficiency of BMP target gene delivery to endogenous stem cells and induce effective tissue regeneration and repair [28]. However, this study only took advantage of ultrasonic triggering in the efficiency of gene transfection, and the implantation of gelatin scaffolder caused greater trauma to the body [29,30]. However, the hydrogel has no therapeutic effect, making it difficult to promote tissue reconstruction through gene therapy. Therefore, although ultrasound technology combined with gene therapy can have a lot of advantages in tissue regeneration, existing studies on ultrasound-triggered biomaterials combined with genetic engineering to promote bone regeneration still have many defects, requiring further research.

Ultrasonic triggering technology combined with genetic engineering to promote tissue regeneration involves in situ regulation of various signaling pathways in tissue cells. There have been studies in which the plasmids were transfected into mouse brains by intraventricular injection of ultrasonic response nanocapsules followed by irradiation using ultrasound [31]. However, this study only explored the successful expression of the transfected protein, but the role and regulation of the target protein on cell and tissue signaling pathways were not clarified. In addition, Yu et al. [32] improved the expression of target genes by using a gene delivery system combined with ultrasound and microvesicles, which played a positive role in the treatment of myocardial infarction. In this study, NF-κB and other signaling pathways were regulated by the introduction of target genes, but no further investigation or explanation was conducted. Wu et al [33] found that targeted cationic microvesicles by ultrasound could effectively deliver plasmids to human retinal endothelial cells and be used for retinal neovascularization in mice. However, in this study, the expression of several key proteins was discussed after plasmid transfection, but a relatively complete cell signaling pathway was not formed, and the mechanism for exploration still needs to be further improved. Therefore, current studies on the mechanism of cell signal transduction promoted by ultrasonic triggering technology combined with genetic engineering are not in-depth and detailed enough. Therefore, it is particularly important to elucidate the cellular signaling mechanism in the study of promoting bone regeneration by genetic engineered ultrasound-triggered biomaterials.

**Fig. 1.**
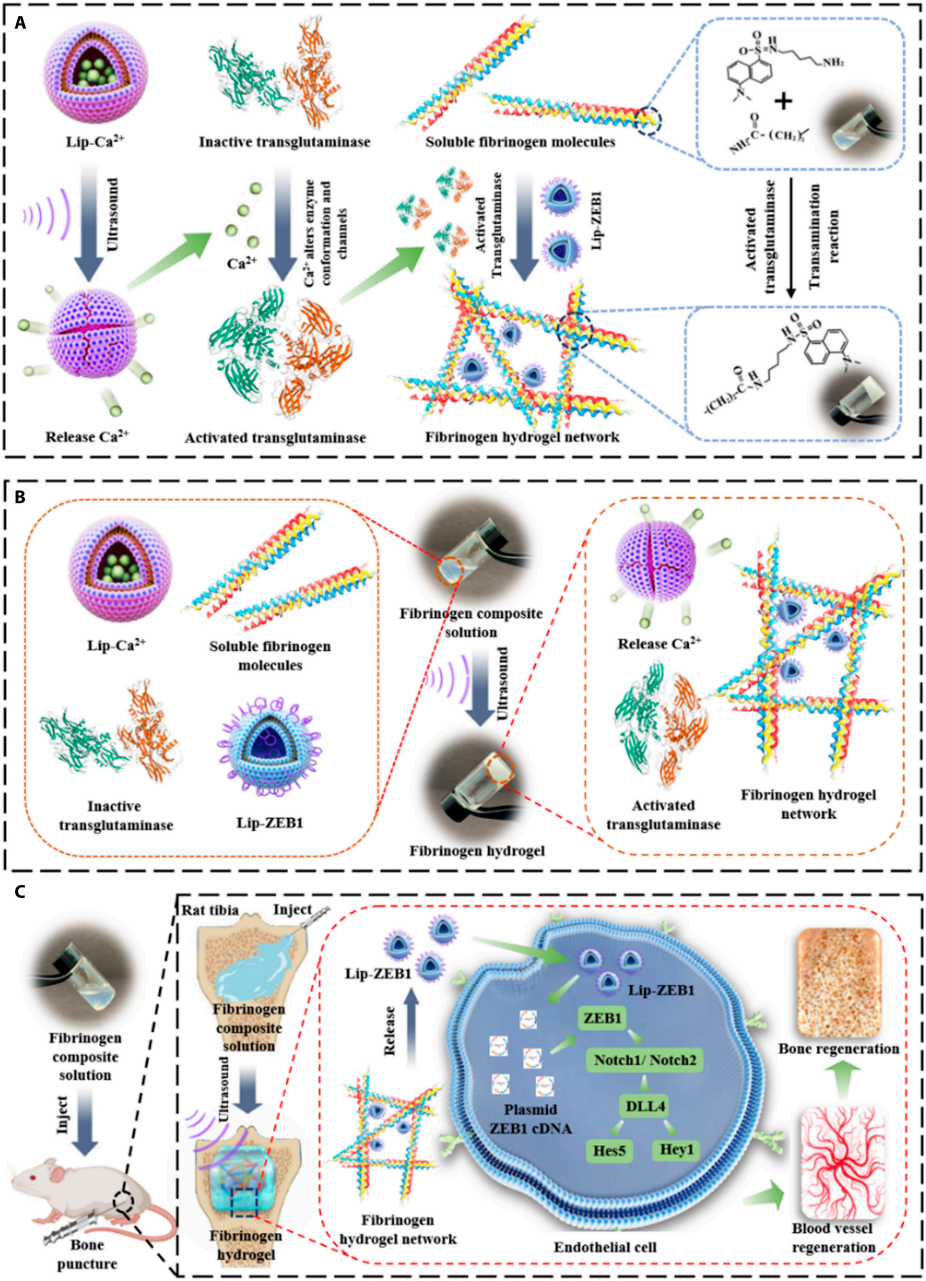
Schematic figure of genetic engineered ultrasonic triggering injectable hydrogel. (A) The liposomes Lip-Ca^2+^ release calcium ions under ultrasonic trigger, and calcium ions activate transglutaminase. Activated transglutaminase catalyzes the transacylation, resulting in covalent cross-linking of lysine and glutamine side-chain residues of fibrinogen molecules, and ultimately triggering the formation of fibrinogen hydrogels. (B) Morphological and structural changes of each component before and after the formation of hydrogel triggered by ultrasound. (C) Genetic engineered ultrasound triggers in situ tibial cross-linking hydrogels to activate angiogenesis through the ZEB1/Notch signaling pathway, thereby promoting bone reconstruction in rats.

Osteoporosis is a bone disease caused by a decline in bone density and bone quality due to a variety of reasons [34,35]. It is particularly important to conduct local bone reconstruction for patients with osteoporosis [36–38]. In this study, for the first time, ultrasound technology was used to construct a genetic engineered hydrogel, in vivo ultrasound-triggered in situ cross-linking was used to achieve cell gene transfection, and finally complete in situ gene therapy was used to promote bone reconstruction. First, under ultrasonic trigger, the liposomes release calcium ion, which changes the energy coordination of transglutaminase, causes the conformational change of transglutaminase, and opens the channel, thus activating transglutaminase. Second, activated transglutaminase catalyzes the transamidation, resulting in covalent cross-linking of lysine and glutamine side-chain residues of fibrinogen molecules, and, at the same time, loading liposome of Zinc-finger E-box-binding homeobox 1 (ZEB1) gene plasmid (Lip-ZEB1), thus triggering the formation of genetic engineered in situ cross-linked injectable hydrogels (Gel-Lip-ZEB1+US). Third, the genetic engineered Gel-Lip-ZEB1+US hydrogel can introduce ZEB1 gene plasmid into endothelial cells through the slow release of Lip-ZEB1, so that the ZEB1 gene can integrate into endothelial cell genome, continuously activate the ZEB1/Notch signaling pathway of endothelial cells, and genetically engineer to promote angiogenesis and bone regeneration. Ultimately, the advanced genetic engineered ultrasound-triggered injectable hydrogel is realized to promote local bone reconstruction of osteoporosis (Fig. 1). Therefore, the advanced genetic engineered ultrasound-triggered injectable hydrogel constructed by us effectively solves many deficiencies of the existing gene therapy to promote tissue reconstruction and has great therapeutic potential in local bone reconstruction of osteoporosis.

## Results

### Preparation and transfection efficiency evaluation of liposome

**Fig. 2.**
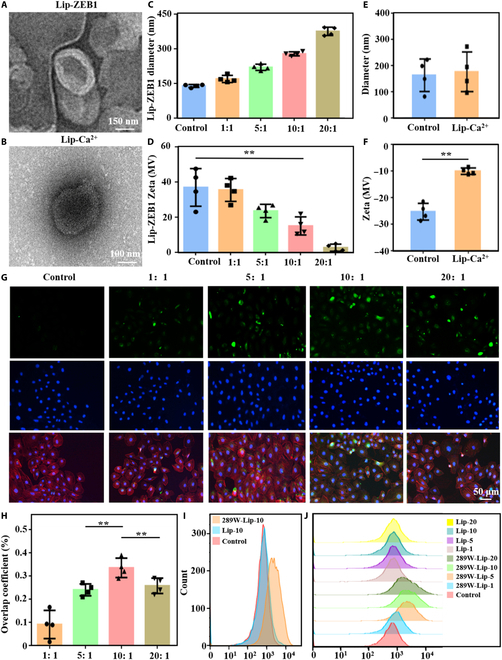
Liposome characterization and transfection efficiency evaluation. (A and B) TEM images of gene-carrying liposomes and calcium-carrying liposomes. (C to F) Potential and particle size characterization of 2 liposomes. (G and H) Fluorescence microscope images and statistical analysis results of different N/P Lip-ZEB1 transfected cells. Green: Target protein fluorescence. Blue: Nucleus. Red: Cytoskeleton. (I and J) Liposomal transfection flow cytometry in the Lip-ZEB1 group and control group. Data are reported as mean ± SD, **P < 0.01.

The mechanism of genetic engineered ultrasound triggering the injectable hydrogel construction process and promoting bone reconstruction is shown in Fig. 1. We prepared 2 kinds of liposomes, Lip-Ca^2+^ directly encapsulated by hydration and 289W transmembrane peptide modified by liposome, to construct 289W-Lip. 289W-Lip loaded the plasmid of the ZEB1 gene through electrostatic adsorption and compression to construct plasmid liposome (Lip-ZEB1). The plasmid structure and gene sequence are shown in Fig. S1. The 2 liposomes showed typical phospholipid bilayer membrane structures by transmission electron microscopy (Fig. 2A and B). Dynamic light scattering results showed that the size and surface charge of liposomes increased with the increase of the proportion of cDNA (Fig. 2C to F).

Fluorescence microscopy showed that different N/P transfected cells had different effects. We observed the expression of green fluorescent protein in cells after 48 h, and quantitatively analyzed the overlap rate of green fluorescence and nuclear blue fluorescence (Fig. 2G and H). The results showed that the fluorescence overlap coefficient was the highest when N/P was 10:1. In addition, it was found via a flow cytometer (Fig. 2I and J) that when N/P was 10:1, the transfection efficiency of the cells was 34.4%. This result was consistent with the above results of fluorescence overlap coefficient rate, and the cell state after transfection is shown in Fig. S2. In addition, we also investigated another liposome, Lip-10, which did not contain the carrier gene of membrane-penetrating peptide. The results showed that the transfection efficiency of the liposome was relatively low with different N/P (Table S1). Therefore, we selected Lip-ZEB1 modified with the 289W transmembrane peptide. Therefore, these experimental results indicated that we have successfully prepared 2 kinds of functional liposomes and can effectively achieve transfection of human umbilical vein endothelial cells (HUVECs).

### Preparation and characterization of genetic engineered injectable hydrogels triggered by ultrasound

**Fig. 3.**
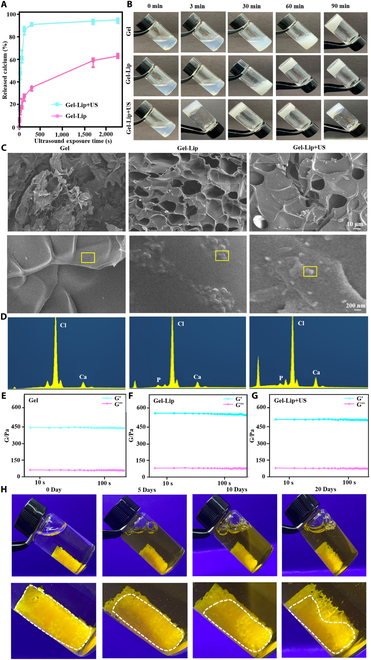
Characterization of genetic engineered injectable hydrogels. (A) Release of calcium ions by liposomes triggered by ultrasound. (B) Cross-linking time and cross-linking status of each hydrogel. (C) SEM images of hydrogels and distribution of surface liposomes. (D) EDS carried out elemental analysis of hydrogel surface conditions. (E to G) Rheological analysis results of Gel, Gel-Lip, and Gel-Lip+US hydrogel. (H). Immersion in a balanced solution after the hydrogel has formed in the artificial bone. The white line is the outline of the hydrogel in the artificial bone.

The formation time of hydrogel, the efficiency of calcium ion release triggered by ultrasound, and the morphology and element composition of hydrogel were very important parameters. Figure 3A shows that the Lip-Ca^2+^ liposome could rapidly release 85% calcium ions within 120 s under ultrasonic trigger. Furthermore, by observing the formation time of hydrogel in different groups (Fig. 3B), compared with the Gel group and the Gel-Lip group, the formation time of hydrogel in the Gel-Lip+US group was the shortest under ultrasonic trigger. The Gel-Lip+US group was found to release calcium ions rapidly within 120 s, activating the interaction between fibrinogen and enzyme. At the same time, hydrogen-bond interactions between liposomes and fibrinogen promoted hydrogel formation [39,40]. The presence of liposomes to some extent promoted hydrogel formation, which was discussed in the Discussion section. Thus, we observed that the Gel-Lip+US group formed a hydrogel at 3 min. However, the Gel-Lip group took nearly 30 min. Thus, these results demonstrated that ultrasound can effectively induce the release of calcium ions in liposomes, thus triggering the formation of hydrogels. Therefore, the ultrasound-triggered hydrogel had strong application prospects in vivo and in vitro. Scanning electron microscopy (SEM) showed that the hydrogel had a porous structure, and the liposome structure on the surface of the hydrogel could be found (Fig. 3C). Energy dispersive spectroscopy (EDS) demonstrated that the surface liposomes of the Gel-Lip and Gel-Lip+US groups contained element P, while the Gel group did not, further confirming the structure of hydrogel-supported liposomes (Fig.3D). The results of rheometer showed that *G*′ (energy storage modulus) and *G*″ (loss modulus) of injectable hydrogels in each group did not change significantly (Fig.3E to G). After pigment staining, the fibrinogen complex solution can be triggered by ultrasound to form in situ hydrogels in the artificial bone and remain stable in phosphate buffer solution (PBS) (Fig.3H). Therefore, we have succeeded in the ultrasound-triggered injectable hydrogel and explored its various properties.

### Transcriptomics of genetic engineered injectable hydrogels on endothelial cells

**Fig. 4.**
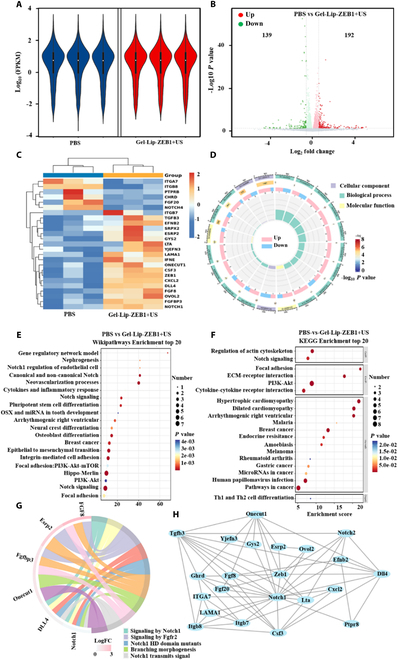
Transcriptomics to investigate the genetic engineering of injectable hydrogels. (A) Violin diagram analysis. (B) Volcano diagram analysis. (C) Heat map analysis. (D) GO enrichment analysis. (E to G) KEGG, Reactome, and Wikipathways Enrichment Analysis. (H) PPI network diagram analysis.

In order to explore the effect of the Gel-Lip-ZEB1+US hydrogel on cell activation at the transcriptional level, the RNA-Seq method was used to obtain the gene expression results of the hydrogel group and the PBS group. Figure 4A shows the overall distribution of gene expression in each group, which was uniform. Compared with the PBS control group, the Gel-Lip-ZEB1+US hydrogel group upregulated 56 differentially expressed genes and downregulated 80 differentially expressed genes (Fig. 4B), including ZEB1, Notch, Dll4, Hes5, Hey1, and other differentially expressed genes. Subsequently, the 25 significantly differentially expressed HUB genes were analyzed by heat map (Fig. 4C) and subsequent series analysis. Gene ontology (GO) enrichment method (Fig. 4D) was used to analyze the function of differentially expressed genes from 3 aspects: biological process, molecular function level, and cell composition. Kyoto Encyclopedia of Genes and Genomes (KEGG), Reactome, and Wikipathways Enrichment analysis (Fig. 4E to G) found that the Notch signaling pathway played a key role in this process (Fig. S3). In addition, the protein–protein interaction (PPI) network map was used to explore the relationship between various HUB genes, and there was strong functional correlation among ZEB1, Notch1, Notch2, Dll4, and other proteins. Based on the above findings, the ZEB1/Notch signaling pathway played an important role in the activation of endothelial cells in the Gel-Lip-ZEB1+US group, which was also consistent with the conclusions of previous studies [41]. We will further verify it in the follow-up experiment.

### Biocompatibility of genetic engineered injectable hydrogels

**Fig. 5.**
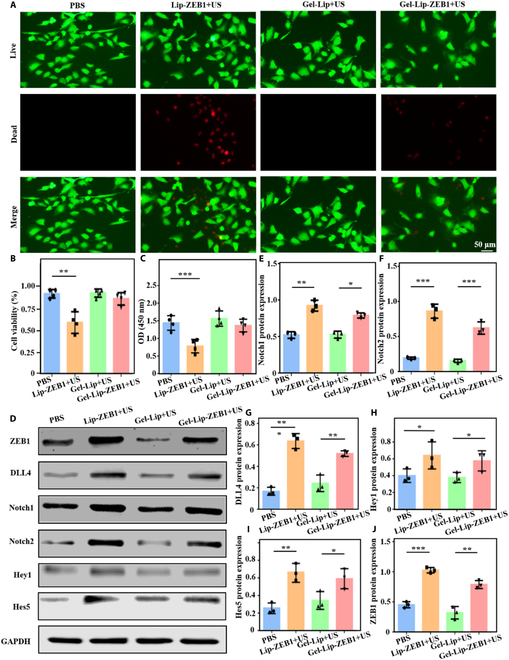
Biocompatibility of genetic engineered injectable hydrogels and gene expression after transfection. (A and B) Live and dead staining figures of endothelial cells in each group and corresponding statistical analysis. (C) Assessment of cell biocompatibility by CCK-8 assay. (D) Figures of Western blotting experiments after cell transfection. (E to J) Quantitative analysis of gene expression was performed. Data are reported as mean ± SD, *P < 0.05, **P < 0.01, and ***P < 0.001.

Biocompatibility is an important parameter for gene cell transfection of biomaterials. Therefore, we evaluated the effects of ultrasound-triggered injectable hydrogels on endothelial cell viability and proliferation using live/dead tests and Cell Counting Kit-8 (CCK-8) tests. Figure 5A and B results show that the cell viability of the hydrogel group and the PBS group was higher, but the efficiency of gene transfection was stronger in the Lip-ZEB1+US group due to the lack of sustained-release effect of liposome. Therefore, it led to a certain degree of biocompatibility decline. In addition, the CCK-8 experiment also showed similar results as the live/dead experiment (Fig. 5C), with stronger cell proliferation in both the hydrogel and PBS groups. These results explained that the genetic engineered injectable hydrogels triggered by ultrasound had good biocompatibility and can be used for further research in vitro and in vivo.

### In vitro transfection of genetic engineered injectable hydrogels

Next, we explored the transfection effect of genetic engineered injectable hydrogel endothelial cells triggered by ultrasound at the cellular level. First, Western blotting experiments explored the protein expressions of key genes (Fig. 5D) and then quantitative analysis of the relative protein expression levels (Fig. 5E to J). Western blotting results showed that compared with the PBS, Lip-ZEB1+US, and Gel-Lip+US groups, the genetic engineered injectable hydrogel Gel-Lip-ZeB1+US could activate ZEB1, Notch1, Notch2, Dll4, Hes5, and Hey1 genes of endothelial cells. It was further proved that Gel-Lip-ZEB1+US can activate the ZEB1/Notch signaling pathway of endothelial cells to play a role in angiogenesis.

**Fig. 6.**
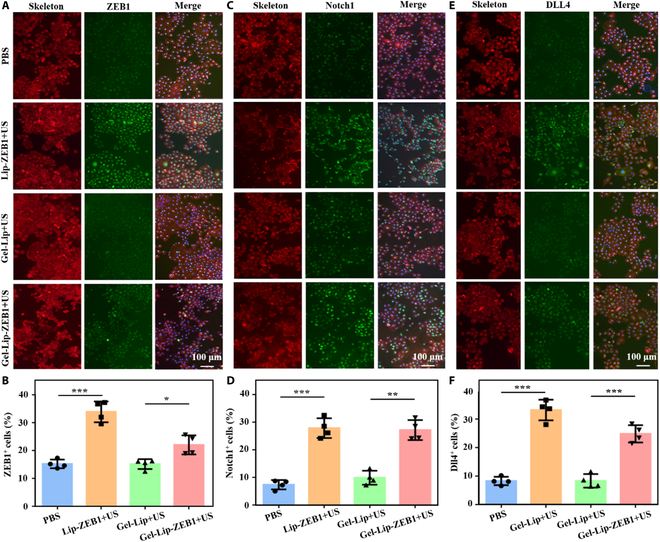
Immunofluorescence results of related gene expression after cell transfection. (A and B) The expression of the ZEB1 gene in transfected cells and the percentage of positive cells were statistically analyzed. (C and D) The expression of the Notch1 gene and the statistical analysis of the percentage of positive cells. (E and F) The expression of Dll4 gene and statistical analysis of the percentage of positive cells. Blue: The nucleus is stained by DAPI. Red: Cytoskeleton stained with phalloidin. Green: Immunofluorescence staining of the target gene. Data are reported as mean ± SD, *P < 0.05, **P < 0.01, and ***P < 0.001.

In addition, we further used cellular immunofluorescence assay to explore the gene transfection effect of hydrogels (Fig. 6A, C, and E). Experimental results showed that compared with the control group, the percentage of ZEB1-, Notch1-, and Dll4-positive cells in the Gel-Lip-ZEB1+US group was significantly higher (Fig. 6B, D, and F). It was worth noting that the gene transfection efficiency of the Lip-ZEB1+US group was significantly stronger, because many genes could be transfected in a short time after direct co-culture of the Lip-ZEB1+US group and endothelial cells. In the Gel-Lip-ZEB1+US group, sustained release of liposomes was achieved, and transfection efficiency was low in a short time. This was also consistent with the experimental results of low cell viability and growth rate of the Lip-ZEB1+US group in the cell compatibility experiment. These results indicated that our genetic engineered ultrasound-triggered injectable hydrogel can achieve good gene transfection effect in vitro and meet the requirements of further in vivo exploration.

### In vivo evaluation of sustained-release properties of genetic engineered injectable hydrogels

**Fig. 7.**
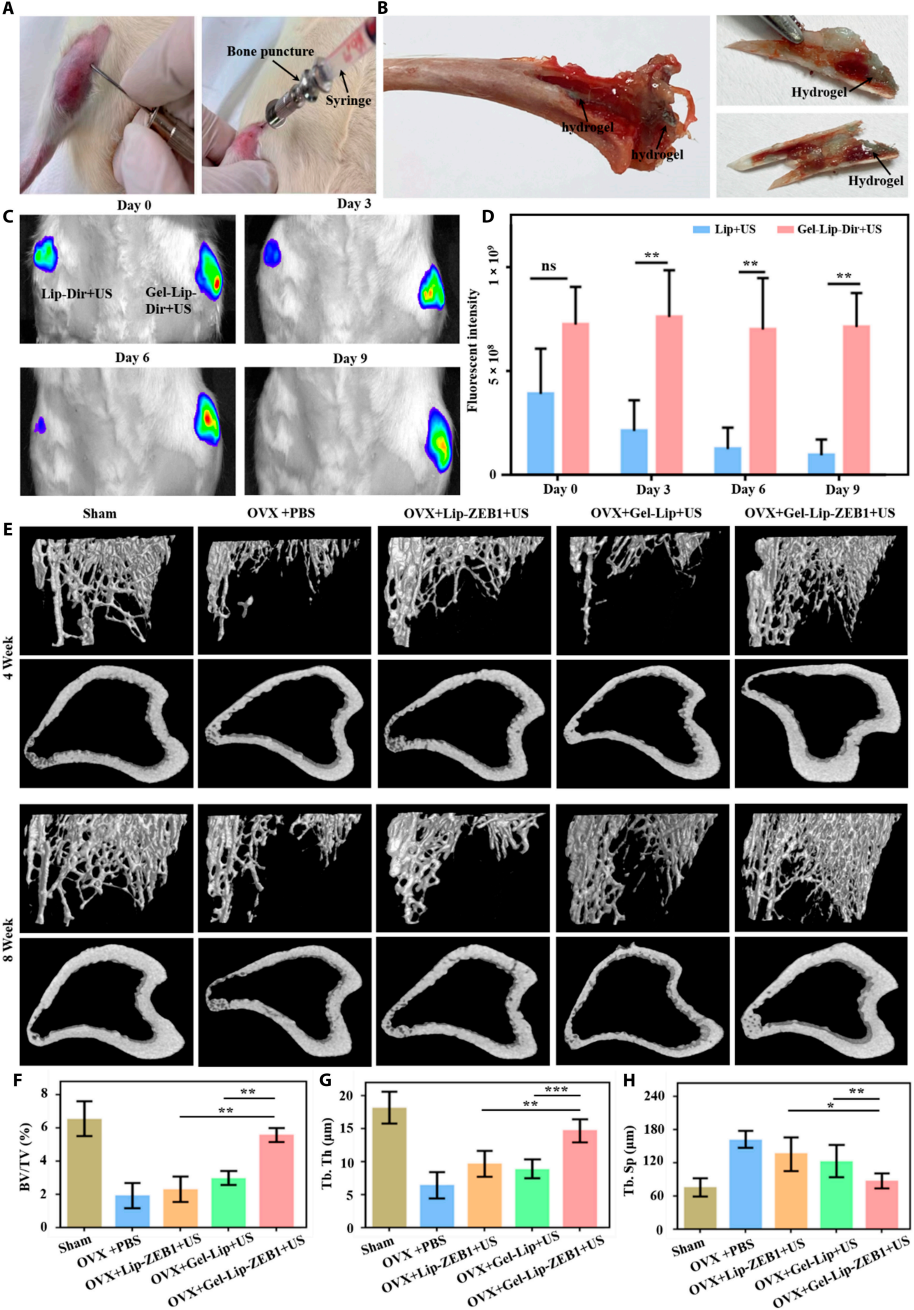
Sustained-released function and bone reconstruction performance of genetic engineered injectable hydrogels. (A) Process diagram of injection of hydrogel into the cancellous bone area of the rat tibia. (B) The genetic engineered hydrogel was formed successfully in tibia. (C and D) Evaluation and quantitative analysis of sustained-released performance of hydrogel loaded liposome. (E to H) Micro-CT evaluation of bone reconstruction in osteoporosis rats and the results of BV/TV, Tb.Th, and Tb.Sp quantitative analysis. Data are reported as mean ± SD, *P < 0.05, **P < 0.01, and ***P < 0.001.

In the study of ultrasound triggering the formation of genetic engineered injectable hydrogels, we used clinical bone-piercing needles. In our study, we found that the instrument could puncture the bone region of the rat and achieve effective injection of the fibrinogen complex solution (Fig. 7A). In addition, using commonly used artificial bones in the clinic, we found that the composite solution of fibrinogen rapidly penetrated the artificial bones (Fig. S4) and formed a stable hydrogel structure under ultrasonic trigger. Notably, we dissected the tibia of the rats after ultrasound and observed the formation of genetic engineered hydrogels triggered by ultrasound. This was also similar to approach used in some previous studies and clinical treatments, which we described in the Discussion section (Fig. 7B). Furthermore, we labeled the liposomes with Dir and formed Gel-Lip-Dir+US hydrogels in rats under the hypergenic trigger. The experimental results showed that the liposomes had completely disappeared 9 days after injection in the Lip-Dir+US group (Fig. 7C and D). Therefore, compared with the Lip-Dir+US control group, the hydrogel group achieved a longer sustained release of liposomes, and the degradation rate was slower in vivo.

### In vivo bone reconstruction of genetic engineered injectable hydrogels

It was found that the genetic engineered injectable hydrogels triggered by ultrasound could achieve good transfection efficiency and good biocompatibility in vitro. Thus, we further evaluated bone tissue formation at 4 and 8 weeks after implantation in the tibial bone. First, we evaluated the in vivo toxic effects of hydrogel implantation on the heart, liver, spleen, lungs, and kidneys 8 weeks after implantation. As can be seen from Fig. S5, no obvious toxic and side effects occurred in the vital organs in the body, and the biocompatibility was relatively good. Micro-CT 3D reconstruction was used to evaluate the effect of bone reconstruction on bone of tibia [42]. The results showed that compared with other groups, the Gel-Lip-ZEB1+US group had the most significant bone regeneration effect (Fig. 7E). There was no significant difference in bone regeneration effect between the Gel-Lip+US group and the PBS group, which was consistent with the above in vivo sustained-release results. Quantitative analysis further confirmed that Bone tissue volume/total tissue volume (Bv/Tv), trabecular thickness (Tb.Th), and trabecular separation (Tb.Sp), key indicators of bone regeneration, were significantly higher than those of other experimental groups and control groups (Fig. 7F to H).

**Fig. 8.**
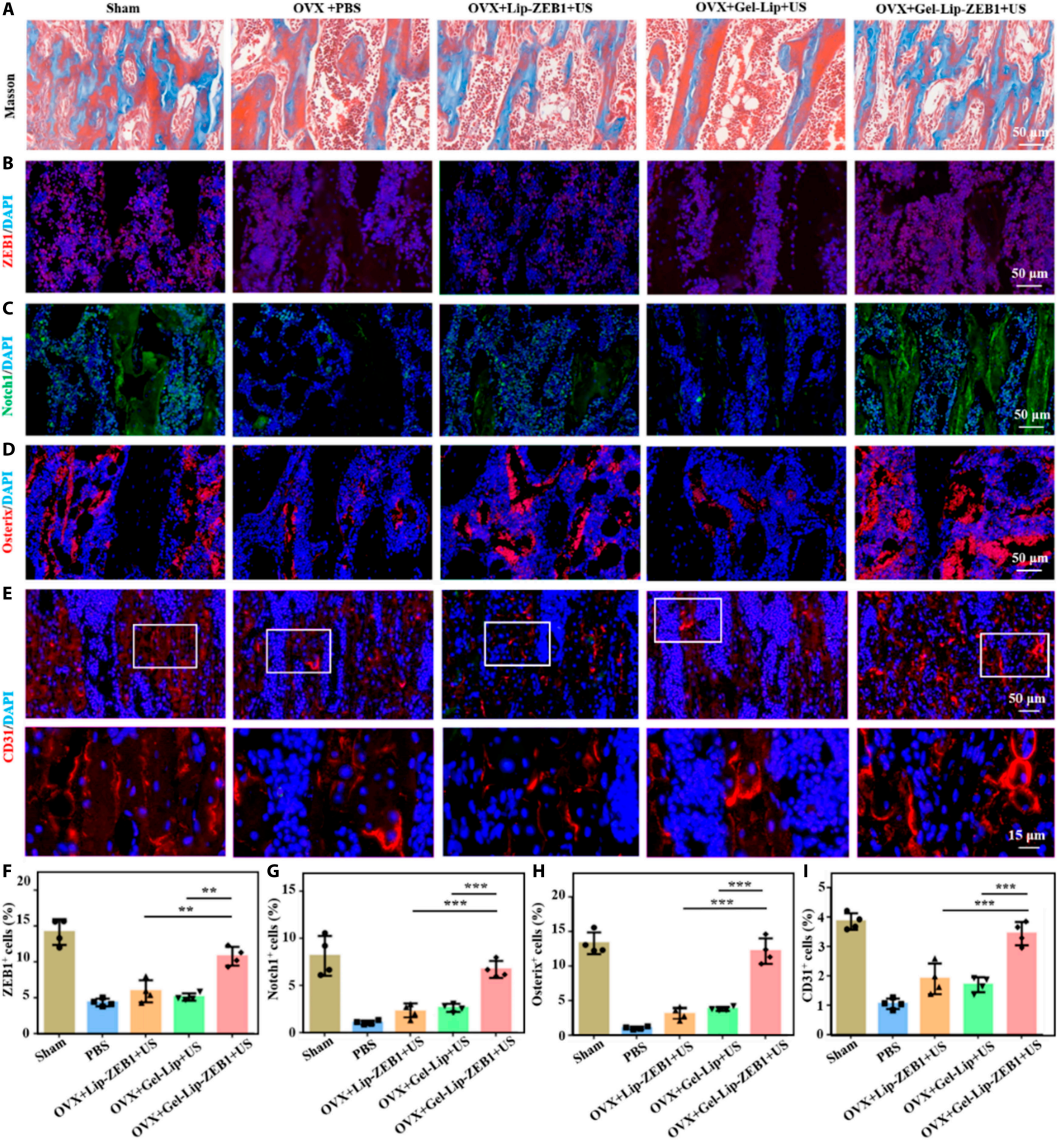
In vivo transfection effect and bone remodeling performance of genetic engineered injectable hydrogels. (A) Masson staining was used to evaluate the regeneration of bone tissue. (B and F) Expression of ZEB1 gene in endothelial cells and its correlation quantitative analysis. (C and G) Expression of Notch1 gene in cells and statistical analysis. (D and H) Expression and statistical analysis of osteogenic genes in vivo. (E and I) CD31 was used to evaluate the situation of vascular regeneration in bone tissue, and corresponding statistical analysis was conducted. Data are reported as mean ± SD, *P < 0.05, **P < 0.01, and ***P < 0.001.

Furthermore, we continued to evaluate the in vivo transfection effect of hydrogels and the effect of bone reconstruction at the histological level. Masson staining showed that after 8 weeks of treatment, there was a significant increase in new bone tissue in the Gel-Lip-ZEB1+US group, and the repair process was gradually completed (Fig. 8A). In addition, according to immunofluorescence staining, ZEB1 and Notch1 were also significantly activated in vivo (Fig. 8B and C). However, there was no significant difference in gene expression between the Gel-Lip+US group and the PBS group (Fig. 8F and G). The effective transfection and slow release of hydrogels were proved. Compared with the other groups, the bone-critical protein Osterix composed of Gel-Lip-ZEB1+US was also significantly increased (Fig. 8D and H). This was because the activation of the ZEB1/Notch signaling pathway indirectly promoted the activation of osteogenic pathway, which will also be elaborated in the Discussion section. Therefore, the genetic engineered ultrasound-triggered injectable hydrogel can achieve good endosomal transfection effect and significantly promote bone regeneration.

### In vivo vascular regeneration of genetic engineered injectable hydrogels

In the above studies, we revealed the good gene transfection effect of hydrogels, which achieved good activation of the ZEB1/Notch signaling pathway in vivo and promoted bone regeneration. Next, we further explored the mechanism by which the genetic engineered ultrasound triggers the hydrogel to promote bone regeneration in vivo. The ZEB1/Notch signaling pathway was mainly involved in bone formation through the activation of endothelial cells and the realization of angiogenesis. First, we evaluated the overall effect of bone reconstruction in each group by hematoxylin and eosin (H&E) staining (Fig. S6). In addition, we evaluated the in vivo angiogenesis effect of hydrogels using immunofluorescence staining of CD31 and VEGF (Fig. 8E and I). Experimental results showed that compared with other groups, the Gel-Lip-ZEB1+US group significantly promoted angiogenesis (*P* < 0.001). Therefore, genetic engineered ultrasound-triggered injectable hydrogels can significantly promote angiogenesis and promote osteogenesis in vivo, thereby improving bone loss.

## Discussion

In the clinical treatment of osteoporosis, systemic treatment and local treatment are commonly used to achieve the therapeutic purpose. Systemic treatment is mainly based on systemic drug intervention, including calcium, calcitriol, hormone replacement, alendronate sodium, parathyroid hormone, and RANKL (receptor activator of NF-κB ligand) inhibitors [43]. However, systemic drug therapy is difficult to sustain long-term release, bone targeting is poor, and repeated administration is required to achieve therapeutic blood drug concentration, which will lead to greater drug side effects and even excessive metabolic load of liver and kidney. In addition, in the population of osteoporosis patients, local bone reconstruction should be promoted early for the femoral neck and other parts that are prone to fracture injury [44,45]. In clinical treatment for osteoporotic fracture patients, due to local cortical bone thinning and bone density reduction, external fixation stent, plate, and screw often encounter a series of problems such as fixation instability, dislocation, and even broken nails. Therefore, in order to solve the above clinical problems, it is particularly important to carry out local bone reconstruction for patients with osteoporosis.

The rigor and feasibility of animal models are an important basis for in vivo investigation and validation of therapeutic effects. In recent years, some scientific research and clinical devices have been used in local bone reconstruction. Yao et al. and Zhou et al. [46,47] show that minimally invasive injection of various biological materials into the tibia of osteoporotic mice has achieved good therapeutic effect on local bone reconstruction. In addition, Arrow EZ-IO and Shusuda have been marketed in clinical treatment and are used for rapid injection of various therapeutic agents into the tibia. In our study, due to the thick cortical bone of rats, it was difficult for the syringe to penetrate directly, so we used the bone-piercing needle commonly used in clinical practice and then injected them with the syringe. The principle was consistent with the above-mentioned research and clinical instruments. Therefore, it is feasible for us to inject the genetic engineered hydrogel triggered by ultrasound into the tibia to achieve local bone reconstruction of osteoporosis. In addition, the genetic engineered hydrogel can be cross-linked in vivo under ultrasonic trigger, and the fluorescence intensity can be stable for a long time, and the degradation rate is slow in vivo. It is worth noting that the hydrogel was cross-linked in the artificial bone and placed in PBS. After degradation for up to 3 weeks, more hydrogel remained in the artificial bone. Therefore, the genetic engineered hydrogel can achieve slow degradation and play a more sustained therapeutic effect.

The ZEB1 gene plays a very important role in the occurrence and development of osteoporosis. We constructed an advanced genetic engineered ultrasound-triggered injectable hydrogel to promote local bone reconstruction of osteoporosis. Through the sustained release of Lip-ZEB1, the genetic engineered injectable hydrogel triggered by ultrasound can introduce the ZEB1 gene plasmid into endothelial cells, integrating the ZEB1 gene into the endothelial cell genome and then activating the ZEB1/Notch signaling pathway in endothelial cells to promote angiogenesis and bone regeneration. Therefore, the ultrasound-triggered genetic engineered injectable hydrogels can achieve gene therapy and bone reconstruction, which is closely related to the ZEB1/Notch signaling pathway. ZEB1 has recently been shown to be able to control bone metabolism through the CD31 subgroup[48,49]. It was found that ZEB1 was highly expressed in CD31 endothelial cells in human and mouse tibia. The knockdown or deletion of the ZEB1 gene leads to the loss of ZEB1 in endothelial cells, which, in turn, leads to the abnormal formation of blood vessels in bone, leading to the obstruction of bone formation [50,51]. Ultrasound-triggered genetic engineered injectable hydrogels affect the Notch signaling pathway epigenetically by overexpressing ZEB1 in endothelial cells and then acting on histone acetylation of Notch1 and DLL4 promoter. However, the Notch signaling pathway is a key signaling pathway for bone formation and angiogenesis. In addition, ZEB1 expression in endothelial cells was significantly decreased in osteoporosis models. Therefore, in our study, sustained transfection of the ZEB1 gene into endothelial cells can be achieved by genetic engineered ultrasound-triggered injectable hydrogels, thus restoring Notch activity of bone endothelial cells, thereby promoting angiogenesis and bone regeneration through the ZEB1/Notch signaling pathway, and ultimately promoting local bone reconstruction of osteoporosis.

The transfection efficiency of liposome is an important parameter in gene transfection of genetic engineered injectable hydrogels triggered by ultrasound [52–54]. When using liposome for gene delivery, the liposome vector has many advantages such as superior genome safety, negligible immunogenicity, and good biocompatibility [55–57]. However, the limited transfection rate and low encapsulation rate hinder its widespread use in gene therapy [58]. Therefore, ultrasound-triggered genetic engineered injectable hydrogels were loaded with 2 cDNA-carrying liposomes, the 289W modified liposome Lip-ZEB1 and the ordinary liposome Lip-10. It was found that the transfection efficiency of 289W modified liposomes was significantly increased to 34.4% at 10:1 N/P. The results are like those of previous studies. Trabulo et al. [59] evaluated the transfection potential of transmembrane peptide-mediated cDNA for cells and obtained a high transfection efficiency of nearly 50% when the peptide–DNA charge ratio was 10:1. Therefore, the ultrasound-triggered genetic engineered injectable hydrogel sustained-release liposomes with transmembrane peptides can effectively transfect cells and complete bone reconstruction. Notably, the in vitro experiments, transfection efficiency, and in vitro effects of the Lip-ZEB1+US group were the best. This is because without the slow-release effect of hydrogel, more ZEB1-loaded liposomes are transfected into cells in a short time. Therefore, the treatment effect of the Lip-ZEB1+US group is better than that of other groups.

The physicochemical properties of the sustained-release liposomes of genetic engineered injectable hydrogels triggered by ultrasound are extremely important [60]. In the characterization of Lip-ZEB1 liposomes, the particle size and surface charge of liposomes increased with the increase of cDNA proportion. This is due to electrostatic adsorption between the positively charged liposome and the negatively charged cDNA, which causes the loaded cDNA to be electrostatic adsorbed to the cationic liposome [61,62]. Thus, the size of the liposomes is increased due to cDNA loading, and the surface charge is decreased, as is the electrostatic interaction between the cationic liposomes and the negatively charged cell membrane. In addition, the presence of liposomes promoted the formation of genetic engineered injectable hydrogels triggered by ultrasound to some extent. It is worth noting that liposomes can be bonded to hydrogel networks through covalent and non-covalent bonds such as hydrogen bonds, making liposomes act as cross-linking agents to some extent and shorten the cross-linking time of hydrogel without the need for interface separation [39,63,64]. Therefore, our study also found that the addition of liposome further shortened the cross-linking time of genetic engineered injectable hydrogels triggered by ultrasound.

In conclusion, the genetic engineering injectable hydrogel was constructed by ultrasonic technology for the first time to realize in vivo ultrasound-triggered in situ cross-linking and cell gene transfection, and finally complete in situ gene therapy to promote bone reconstruction. This hydrogel can introduce ZEB1 gene plasmid into endothelial cells by releasing Lip-ZEB1, so that the ZEB1 gene can be integrated into the genome of endothelial cells, thereby activating the ZEB1/Notch signal pathway of endothelial cells, promoting vascular regeneration and bone regeneration, and ultimately promoting the local bone reconstruction of osteoporosis by genetic engineering. Therefore, our genetic engineered ultrasound-triggered injectable hydrogel can be used as a promising triggered genetic engineering biomaterial to provide a new treatment method for osteoporosis.

## Materials and Methods

### Materials

The following materials were used in this study: lecithin (Macklin, China); cholesterol (Aladdin, China); 1,2-dioleoyl-sn-glycero-3-phosphoethanolamine (DOPE, Highfine, China); DSPE-mPEG (1,2-distearoyl-sn-glycero-3-phosphoethanolamine-*N*-[methoxy(polyethylene glycol)]) (shyuanye, China); chloroform (Aladdin, China); 2,3-dioleoyloxy-propyl-trimethylammonium-chloride (DOTAP, Pharmaceutical Tech, China); transglutaminase (shyuanye, China); fibrinogen (Yeasen, China); Calcium Ion Color Development Kit (Beyotime, China); 4',6-diamidino-2-phenylindole (DAPI) (Solarbio, China); artificial bone (Mindray, China); calcium chloride (Aladdin, China); CCK-8 Kit (Servicebio, China); Live/Dead Cell Kit (Servicebio, China); radioimmunoprecipitation assay (RIPA) lysate (Beyotime, China); and bicinchoninic acid (BCA) protein kit (Beyotime, China).

### Preparation and characterization of liposomes

The function of the first kind of liposome is mainly Ca^2+^ loading (Lip-Ca^2+^). In simple terms, lecithin/cholesterol/mPEG-DSPE (52:43:5, mol/mol/mol) was weighed and dissolved in l ml of chloroform (Aladdin, China) at 38 °C, vacuumed with a rotary evaporator, and dried. Next, the dried lipid membrane was hydrated by adding 20 mg/ml CaCl_2_ solution and 10% energy of ultrasonic cell breaker for 10 min to obtain dispersed multilayer liposomes. Impurities in the liposome solution were then removed through 0.45- and 0.22-μm membrane filters (Irish Milex). Next, calcium-laden liposomes were dialyzed with an isotonic buffer (0.6 M sodium chloride) to remove free calcium and stored at 4 °C.

The main function of the second type of liposome was load gene. Firstly, 289W-PEG-DSPE was prepared by Michael addition reaction. The preparation process was as follows: DSPE-PEG-Mal was dissolved in DMF, and CYS-labeled 289W polypeptide (Apeptide, Shanghai, China) was dissolved in PBS, mixed with a molar ratio of 1:1.2, stirred at room temperature for 4 h, and then dialyzed with 3.5-kDa dialysis bags to remove excess 289W. Purified 289W-PEG-DSPE was obtained after freeze-drying. Next, DOPE, DOTAP, cholesterol, and 289W-PEG-DSPE (40:30:30:2, mol/mol/mol/mol) were weighed in proportion and dissolved in l ml of chloroform. At 60 °C, a rotary evaporator was used to vacuum and dry. Next, ultrasound conditions such as Lip-Ca^2+^ liposomes were used to obtain dispersed multilayered liposomes. The filter was also filtered with 2 different membrane diameters and then stored at 4 °C. In addition, liposome without 289W was prepared as control, and the rest of the preparation process was the same as above.

The diameter and morphology of the liposomes were observed using transmission electron microscopy (Thermofisher, USA). In addition, the liposome sample was diluted in an isotonic buffer and the liposome particle size and zeta potential were measured by dynamic light scattering using a Zetasizer (Malvern Nano-ZS, UK) instrument.

### DNA-packaged liposome preparation and transfection efficiency evaluation

HUVECs were cultured in 24-well plates to achieve a fusion rate of about 70%, and washed with buffer solution for 3 times. We select the second liposome from the previous section for further manipulation. Plasmid:Liposome = 1:1, 5:1, 10:1, 20:1. The plasmid-free group was the control group. According to the above transfection ratio, plasmids and lipid body fluids were diluted with 50 μM serum-free medium, respectively. After successful configuration, the 2 reagents were mixed for 30 min, then added to serum-free medium and cell co-culture for 6 h, and then added to serum-containing medium for 48 h. The plasmid containing the ZEB1 gene was co-incubated with liposome and became Lip-ZEB1-289W, which was hereinafter referred to as Lip-ZEB1 for short. After culture, the cells were washed 3 times with buffer. According to the instructions, the cells were stained with DAPI. After staining, the HUVECs were washed 3 times with phosphate buffer. Fluorescence images of HUVECs were observed under a fluorescence microscope (Nikon, Japan), and the transfection efficiency was determined by observing the fluorescence intensity of each group. The fluorescence colocalization repetition rate was analyzed and the transfection rate was evaluated using ImageJ 1.8.0 software. In addition, the transfection efficiency of the cells was measured by flow cytometry (Cytek, USA) after transfection.

### Ultrasound-triggered release of calcium ions and preparation of genetic engineered injectable hydrogels

Ultrasonic therapy instrument (Dimip, China) with a frequency of 1 MHz and an intensity of 3 W was used. Ultrasound was applied to 250 μl of calcium ion liposomes for 0 to 2,400 s. According to the instructions, the release concentration of calcium was measured with a calcium ion color development kit, and the release curve was plotted using GraphPad Prism 9. Fibrinogen was dissolved with 0.9% NaCl and the final concentration was 22.42 mg/L, and then mixed with the above 250 μl of calcium-carrying liposome and the final concentration of 8.69 mM transglutaminase to prepare the fibrinogen complex solution. After ultrasonic treatment for 100 s with ultrasonic probe, the formation time of hydrogel was observed, and the Gel-Lip+US hydrogel was obtained. If the fibrinogen complex solution had no ultrasonic effect, it is the Gel-Lip group. In addition, 22.42 mg/L of fibrinogen and 8.69 mM of transglutaminase and calcium were mixed to form the Gel group.

### Characterization of ultrasound-triggered genetic engineered injectable hydrogels

The hydrogel modulus was analyzed by a HAAKE MARS III rheometer at 37 °C. The hydrogel modulus was monitored over time by a rheometer with a parallel geometry of 20 mm. In addition, SEM (FEI, USA) and x-ray EDS were used to study the morphology of hydrogels and the loading of liposomes. In addition, after staining, the fibrinogen complex solution was dropped into the artificial bone to observe the permeability of the solution in the artificial bone and the state of the hydrogel in the buffer for 0, 5, 10, and 20 days.

### Transcriptome analysis

The cells were transfected and transcriptome analysis was performed. Total RNA was extracted using Trizol (Beyotime, China) according to the instructions. NanoDrop 2000c (Temo, USA) can be used to explore RNA concentration and Agilent 4100 Bioanalyzer (Agilent, USA) to detect quality. Transcriptome sequencing was performed by Shanghai OE Biotech. Co. (Shanghai, China). For transcriptome results, R software was used to analyze differentially expressed genes (DEGs), KEGG, and GO. *P* < 0.05 and fold change > 2 or fold change < 0.5 were used as the threshold of differential expression.

### Evaluation of cellular biocompatibility of genetic engineered injectable hydrogels

PBS buffer was added to the control group. The Gel-Lip-ZEB1+US group was an injectable hydrogel triggered by ultrasound loaded with Lip-ZEB1, the Lip-ZEB1+US group was hydrogel free, and the Gel-Lip+US group had no ZEB1 plasmid. Lip-ZEB1+US, Gel-Lip+US, and Gel-Lip-ZEB1+US were treated with ultrasound (frequency, 1 MHz; intensity, 1 W; 100 s), and the formation of hydrogel was observed. The materials and cells of each group were co-cultured using a Transwell plate. CCK-8 detection: According to the instructions, the CCK-8 kit was used to detect the proliferation and cytotoxicity of hydrogels on HUVECs. After transfection for 48 h, cells of each group were incubated with reagents. Finally, absorbance was determined at 450 nm wavelength. In addition, live/dead cell kits were used to test the activity of HUVECs. After incubation according to the instructions, a fluorescence microscope (Nikon, Japan) was used to take live/dead fluorescence images of the cells. The red fluorescence was produced by propyl iodide, which represented dead cells. Calevin-AM can stain living cells and show green fluorescence [65].

### Western blot and cell immunofluorescence

The expression of genes related to the ZEB1/Notch signaling pathway was detected by Western blotting. We used a Transwell plate to co-culture materials and endothelial cells in each group. According to the instructions, RIPA lysate was used to split the cells of each group. The total protein concentration was detected by the BCA protein kit. During the Western blot process, related primary antibodies included ZEB1 (abcam, UK, 1:500), Dll4 (abcam, UK, 1:500), Notch1 (abcam, UK, 1:1,500), Notch2 (abcam, UK, 1:1,000), Hey1 (abcam, UK, 1:1,500), Hes5 (abcam, UK, 1:5,000), and GAPDH (abcam, UK, 1:2,500). Secondary antibodies of the corresponding species were incubated. In addition, a chemiluminescence system was used for detection and imaging. Finally, the Western blotting results were quantitatively analyzed by ImageJ software [66].

In addition, immunofluorescence staining was used to further analyze the expression of related genes. After cell transfection, cells were fixed with 4% paraformaldehyde (Beyotime, China). Next, cells were permeated using 0.1% Triton X-100 and washed with PBS. Primary antibody was diluted with diluting solution and incubated overnight at 4 °C. Primary antibody included ZEB1 (abcam, UK, 1:250), Notch1 (abcam, UK, 1:125), and DLL4 (abcam, UK, 1:250). The secondary antibodies of the species used were used for incubation. Fluorescence microscopy (Nikon, Japan) was used to take immunofluorescence images of cells. The results of Western blotting were quantitatively analyzed by ImageJ software.

### Establishment of an osteoporosis model and evaluation of genetic engineered injectable hydrogels for bone reconstruction

As mentioned in the previous article, an animal model of osteoporosis was established [67]. Next, Sprague–Dawley rats were anesthetized with isoflurane and an animal gas anesthesia machine. A bone-piercing needle was used to puncture the bone area of the metaphysis of the tibia, and the solution of each group was injected. Since the bone-piercing needle has penetrated the tibial cortical bone region, the hydrogel precursor solution can self-penetrate the spongy bone region of the tibia in order to form a hydrogel in the tibia region for therapeutic purposes. In recent years, some studies have realized the therapeutic effect of local bone reconstruction by minimally invasive injection of various biological materials into the tibia of osteoporosis mice [46,47]. In addition, Arrow EZ-IO and Shusuda have been marketed in clinical treatment and were used for rapid injection of various therapeutic drug reagents into the tibia. We also described it in the Discussion section. Therefore, it was feasible to form in situ ultrasound-triggered genetic engineered hydrogels in rat tibia to achieve local bone reconstruction in osteoporosis. In addition, in order to further verify that ultrasound can trigger the formation of fibrinogen hydrogel, we dissected rat tibia after ultrasound treatment to further observe the formation of hydrogel in the tibia.

Lip-ZEB1 liposomes were prepared into dir-labeled liposomes, and 100 μl of reaction solution was injected into the articulatory bone region of tibial metaphyseal by a bone-piercing needle. There was no fibrinogen complex solution in the control group. After ultrasound (1 MHz, 1 W, 100 s), observation was performed for 0, 3, 6, and 9 days, and biological imaging analysis was performed using the IVIS Spectrum in Vivo imaging system. Areas of interest were circled in tibia tissue and the fluorescence intensity of dir was measured by Image software. The Sham group was normal rats, and the OVX+PBS group was injected with PBS after successful modeling. OVX+Lip-ZEB1+US, OVX+Gel-Lip+US, and OVX+Gel-Lip-ZEB1+US groups were triggered by ultrasound after injection of the corresponding reaction solution. Twenty rats were randomly divided into 5 groups with 4 rats in each group.

### Micro-CT analysis

The animals were sacrificed at 4 and 8 weeks, respectively, and fresh tibia was dissected and fixed with 4% paraformaldehyde solution. High-resolution micro-CT was used (Belgium, Bruker, Skyscan1076; spatial resolution, 18 μm) to scan the tibial metaphyseal. Bv/Tv, Tb.Th, and Tb.Sp were measured and compared.

### Histological and histological immunofluorescence studies

The tibia samples were stored in 4% paraformaldehyde solution for histological studies (*n* = 4). The tibia was decalcified and processed into 5-μm-thick tibia slices. H&E and Masson staining were used to observe the ability of bone reconstruction at the histological level. CD31 (abcam, UK, 1:1,000), VEGF (novus, USA, 1:250), ZEB1 (abcam, UK, 1:250), Notch1 (abcam, UK, 1:125), and Osterix (abcam, UK, 1:500) were used to detect the performance of vascular remodeling and bone regeneration in tibial tissue. In addition, the heart, liver, spleen, lung, and kidney of rats were also subjected to H&E and the biocompatibility of hydrogel was observed. All H&E staining and Masson staining were observed and photographed with an optical microscope (Nikon, Japan). Immunofluorescence was photographed using a laser scanning confocal microscope (Zeiss, Germany).

## Data Availability

All data needed to evaluate the conclusions in the paper are present in the paper and/or the Supplementary Materials.
