# Supplementary material for: Genetic Engineered Ultrasound-Triggered Injectable Hydrogels for Promoting Bone Reconstruction
Source: Research (Wash D C). 2023 Sep 1;6:0221. doi: 10.34133/research.0221 (PMC11740919; doi:10.34133/research.0221)
Supplement: Supplementary 1 — Figs. S1 to S6 Table S1 [file research.0221.f1.docx]

*·Supplementary Materials*

**Genetic engineered ultrasound-triggered injectable hydrogels**

**for promoting bone reconstruction**

Zhenyu Zhao^1, 2†^, Huitong Ruan^2†^, Aopan Chen^1^, Wei Xiong^3^, Mingzhu Zhang^3*^, Ming Cai^1*^, Wenguo Cui^2⁎^

^1^ Department of Orthopaedics, Shanghai Tenth People's Hospital, School of Medicine, Tongji University, No.301 Middle Yanchang Road, Shanghai 200072, PR China

E -mail: cmdoctor@tongji.edu.cn

^2^ Department of Orthopaedics, Shanghai Key Laboratory for Prevention and Treatment of Bone and Joint Diseases, Shanghai Institute of Traumatology and Orthopaedics, Ruijin Hospital, Shanghai Jiao Tong University School of Medicine, 197 Ruijin 2nd Road, Shanghai 200025, P. R. China.

E -mail: wgcui80@hotmail.com; wgcui@sjtu.edu.cn

^3^ Department of Foot and Ankle Surgery, Beijing Tongren Hospital, Capital Medical University, 1 Dongjiao Minxiang, Beijing, 100730, China.

Email: mingzhuzhang@mail.ccmu.edu.cn

^⁎^Corresponding authors.

E-mail addresses: [mingzhuzhang@mail.ccmu.edu.cn](mailto:mingzhuzhang@mail.ccmu.edu.cn) (M. Zhang)；cmdoctor@tongji.edu.cn (M. Cai); wgcui80@hotmail.com (W. Cui)

^†^These authors contributed equally to this work.


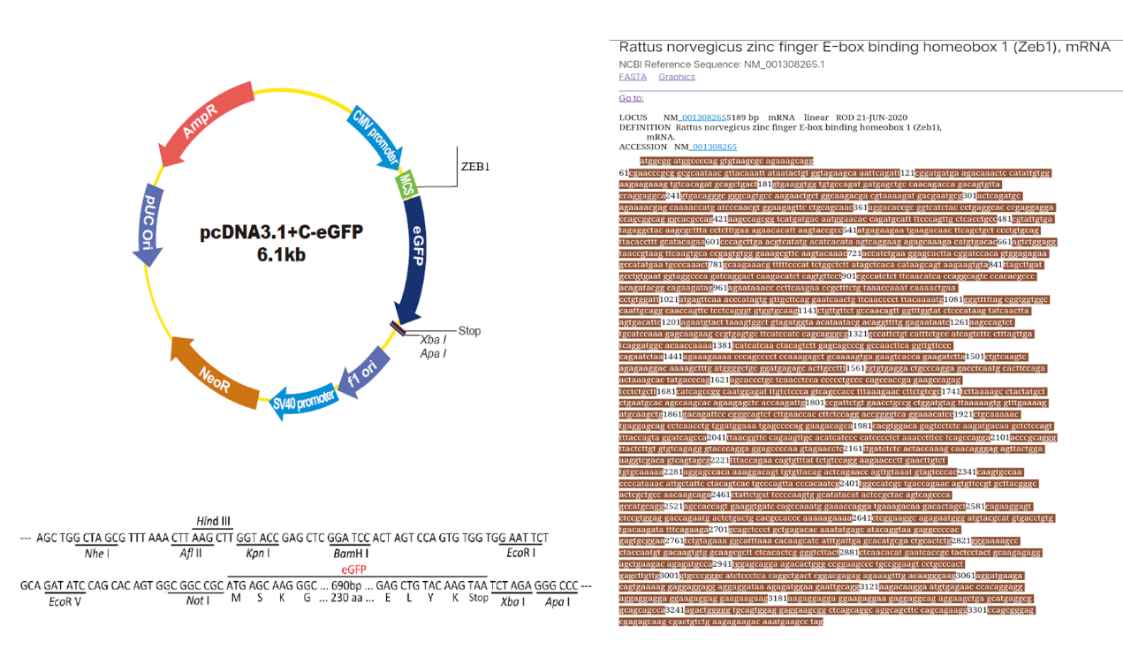


Fig. S1. Plasmid diagram and gene sequence of target gene ZEB1.


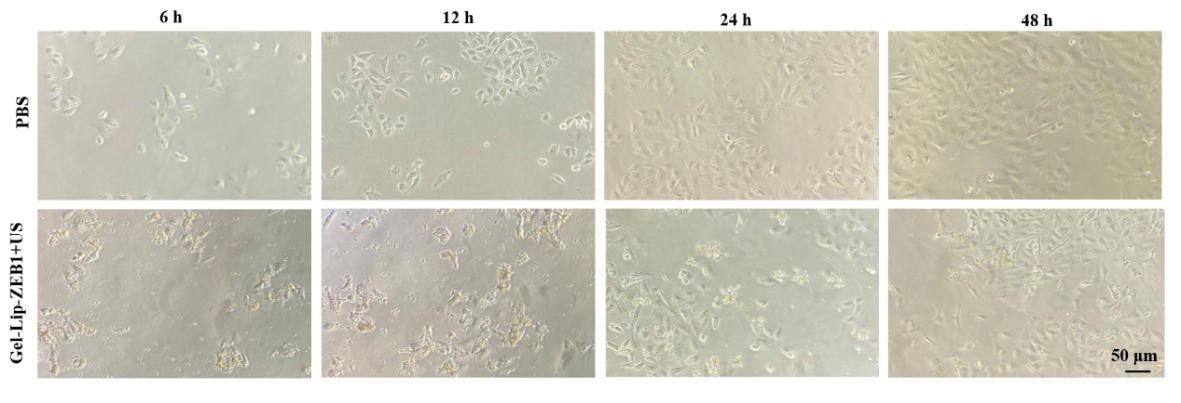


Fig. S2. Morphological changes of genetic engineered injectable hydrogel and endothelial cells after co-culture.


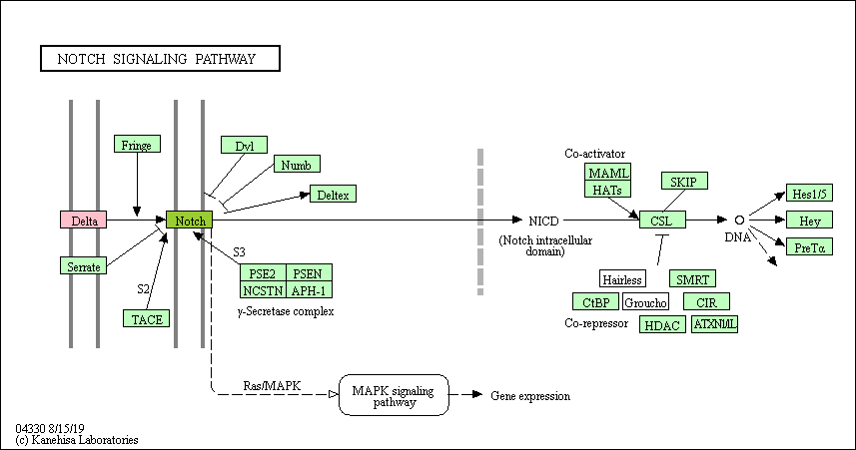


Fig. S3. Transcriptome sequencing after transfection of genetic engineered injectable hydrogel cells showed that Notch signaling pathway plays an important role in this process.


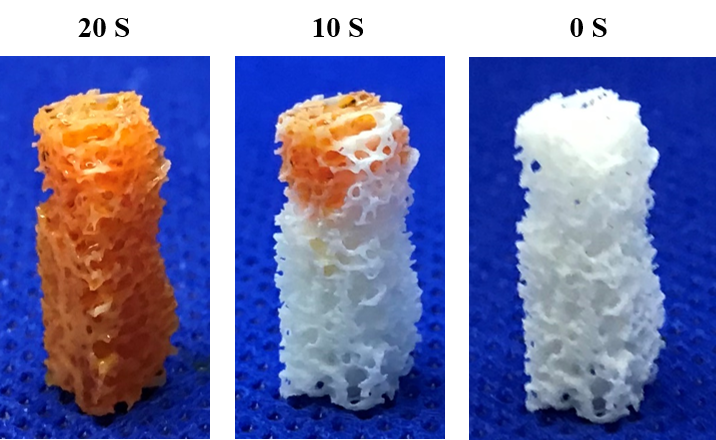


Fig. S4. Evaluation of permeability of stained fibrinogen complex solution in cancellous bone.


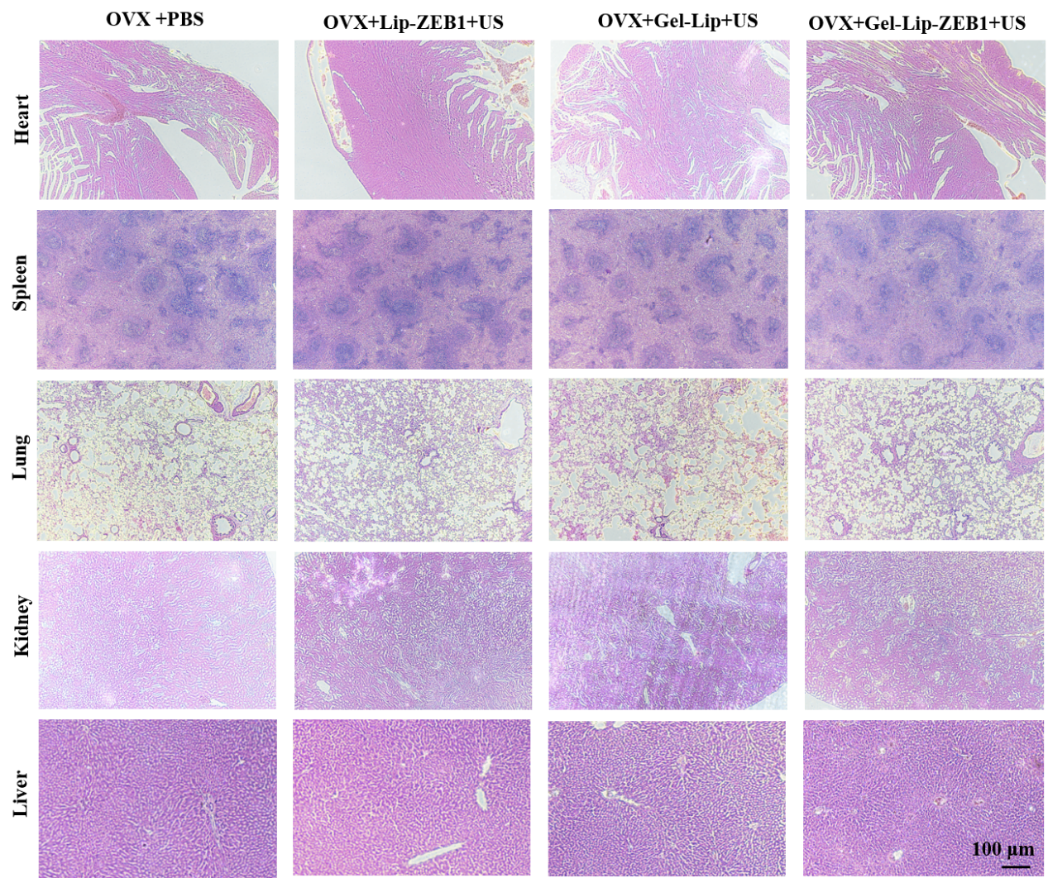


Fig. S5. Evaluation of biocompatibility of genetic engineered injectable hydrogel in vivo.


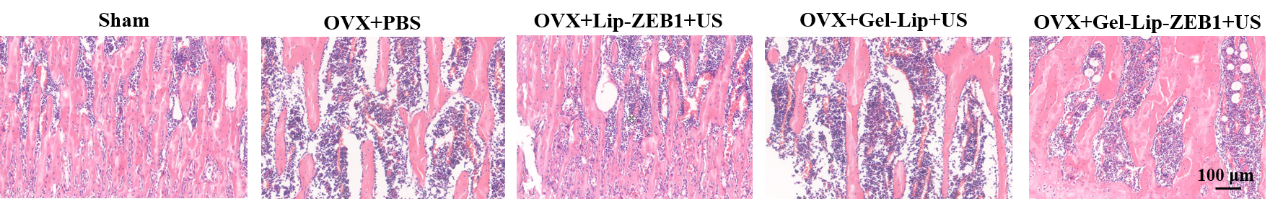


Fig. S6. HE staining was used to evaluate the regeneration of bone tissue.

Table 1: Transfection efficiency of genetic engineered injectable hydrogel cells was measured by flow cytometry.

|  | Blank | Lip-1 | Lip-5 | Lip-10 | Lip-20 | 289W-1 | 289w-5 | 289w-10 | 289w-20 |
| --- | --- | --- | --- | --- | --- | --- | --- | --- | --- |
| Overlap coefficient | 0.43% | 0.6% | 1.55% | 1.34% | 1.43% | 1.44% | 28.4% | 34.4% | 25.9% |
| Mean fluorescence intensity | 654 | 793 | 912 | 775 | 875 | 932 | 2465 | 2854 | 2431 |
